# Acute Kidney Injury and Diffuse Pulmonary Hemorrhage Secondary to IgA Nephropathy and Henoch-Schönlein Purpura: A Case Report

**DOI:** 10.7759/cureus.43054

**Published:** 2023-08-06

**Authors:** Raidah Ayesha Razack, Abubakr O Bajaber, Ahmed Elsharkawy, Ahmad Alghitany

**Affiliations:** 1 College of Medicine, Alfaisal University, Riyadh, SAU; 2 Department of Medicine, Saudi German Hospital, Riyadh, SAU; 3 Department of Nephrology, Ain Shams University, Cairo, EGY

**Keywords:** iga nephropathy, pulmonary-renal syndrome, case report, diffuse pulmonary hemorrhage, acute kidney injury, henoch schönlein purpura

## Abstract

IgA nephropathy (IgAN), characterized as immune complex-mediated glomerulonephritis, can occasionally manifest alongside the pulmonary-renal syndrome. Henoch-Schönlein purpura (HSP), an inflammatory condition affecting small vessels through leukocytoclastic vasculitis, exhibits a close association with IgA nephropathy. Nonetheless, HSP's infrequent complications encompass pulmonary hemorrhage. Notably, the onset of pulmonary hemorrhage can rapidly precipitate a grave decline in the patient's health status, carrying a potentially fatal outcome for both disorders. Moreover, the existing literature regarding this specific complication and its management, particularly among adults, remains relatively limited. We report a rare case of a 43-year-old male with acute renal failure secondary to IgA nephropathy associated with HSP, whose condition was further complicated by pulmonary hemorrhage. He was treated with extensive plasmapheresis, pulse steroids, rituximab, and cyclophosphamide, which led to the successful recovery of his kidney function. Recognizing the potential of various presentations can significantly contribute to early diagnosis and prompt treatment, potentially leading to an improved prognosis for these patients.

## Introduction

IgA nephropathy (IgAN) is an immune complex-mediated glomerulonephritis characterized by diffuse mesangial IgA deposits. The most common presentations of IgAN involve an initial upper respiratory tract infection followed by recurring episodes of hematuria, often accompanied by proteinuria. The prevailing presentation is simultaneous pulmonary and renal involvement, wherein pulmonary hemorrhage stands out as the predominant pulmonary complication [1]. Henoch-Schönlein purpura (HSP) is a transient, acute small-vessel vasculitis commonly observed in children and associated with IgAN. Its hallmark features include distinctive non-thrombocytopenic palpable purpura on the buttocks and lower extremities, along with symptoms of arthralgia and gastrointestinal and renal involvement [2]. While pulmonary hemorrhage is an infrequent occurrence in HSP, when it does manifest, it tends to be severe, often necessitating mechanical ventilation for approximately 50% of affected individuals [3]. Importantly, the presence of pulmonary hemorrhage serves as an adverse prognostic indicator in both IgAN and HSP cases, demanding assertive interventions to mitigate its impact.

This case report introduces a 43-year-old male who sought medical attention at the hospital for abdominal distention, leg swelling, and a purpuric rash observed on the lower extremities. His hospital course was complicated by acute kidney injury, pulmonary hemorrhage, and pancytopenia. The patient's condition showed marked improvement over three months after successful treatment with a combination of cyclophosphamide, rituximab, pulse steroids, and extensive plasmapheresis.

## Case presentation

**Figure 1:**
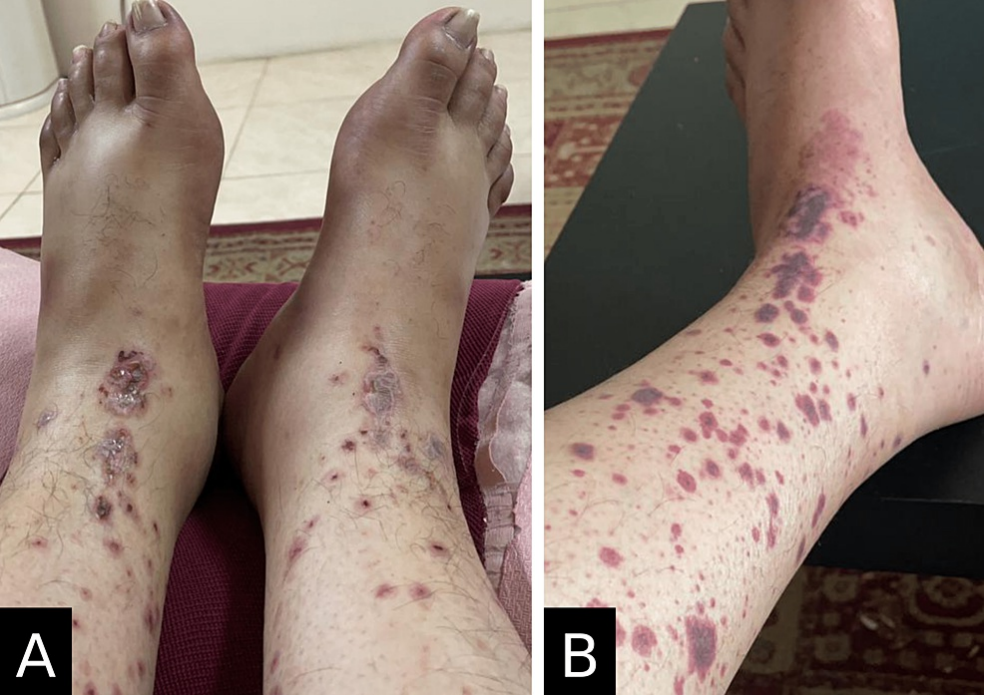
Physical findings (A) Bilateral lower limb pitting (not shown) edema with purpuric rash; (B) scattered purpura over the lower limbs (left leg).

A 43-year-old male was admitted to the hospital with a severe acute kidney injury and purpuric skin eruptions. He had recently been diagnosed with diabetes mellitus (HbA1C: 8.94%) and had no significant family history related to his presentation. On examination, the patient looked toxic and pale but was conscious, alert, and oriented. His vital signs showed a blood pressure of 130/80 mmHg, a pulse rate of 88/min, and a respiratory rate of 22/min; however, he was afebrile. Physical examination revealed mild ascites and generalized anasarca with no organomegaly. Severe bilateral equal pitting edema accompanied by several purpuric eruptions in the lower limbs (Figure 1). The review of other systems was normal.

Upon admission, lab investigations revealed severe hypoalbuminemia (1.9 mg/dL) and nephrotic-range proteinuria (9,400 mg/24 hours). A complete blood count showed leukopenia and neutropenia (white blood cells [WBC]: 2.79 × 10^9^/L), anemia (red blood cells [RBC]: 2.74 × 10^12^/L; hemoglobin: 7.30 g/dL; hematocrit: 22.9%), and thrombocytopenia (platelets: 20 × 10^9^/L). Fibrinogen levels were also found to be reduced. To address this, the patient received transfusions of packed red blood cells, fresh frozen plasma, and cryoprecipitate. Additionally, Filgrastim was administered. Urinalysis revealed trace protein, with erythrocytes at 60-70/hpf and pus cells at 2-3/hpf. Notably, the 24-hour urine protein collection demonstrated a substantial proteinuria level of 9400 mg, indicating nephrotic-range proteinuria. His baseline creatinine was 0.86 mg/dL a month ago.

Multiple imaging studies were done throughout his hospital course to rule out possible underlying causes of his deteriorating kidney function. A duplex Doppler study of the renal arteries revealed a bilaterally increased renal artery resistive index, suggesting advanced kidney disease. An ultrasound showed normal-sized kidneys with no hydronephrosis, mild hepatomegaly, and marked bowel gaseous distension.

Immunological studies showed decreased levels of serum complements C3 and C4, which were 48.4 mg/dL and 17.8 mg/dL, respectively. Serology for antinuclear antibodies (ANA), anti-neutrophil cytoplasmic antibodies, and anti-ds-DNA were negative. The antistreptolysin O (ASO) titer was normal (24 IU/mL). The IgA level was also within the normal range (212 mg/dL).

Besides his daily dialysis for renal failure, the patient was started on alternate-day plasmapheresis due to his rapidly declining kidney functions. He received a 100% replacement of his plasma volume after 10 daily plasmapheresis sessions. He then began 500 mg of IV pulse steroid therapy (solumedrol) for five days to taper it off over the next six months. To correct his hypoalbuminemia and volume overload status, he was started on IV albumin and IV Lasix. In addition to this, he was receiving insulin to control his DM and prophylactic anticoagulants.

On the sixth day of admission, despite all the management protocols, the patient continued to have severe hypoalbuminemia and thereby continued receiving IV albumin and lasix. Anticoagulant therapy had to be discontinued the next day as he had an episode of hematuria. Vitamin K and Irbesartan (300 mg) were added to his treatment regimen.

**Figure 2:**
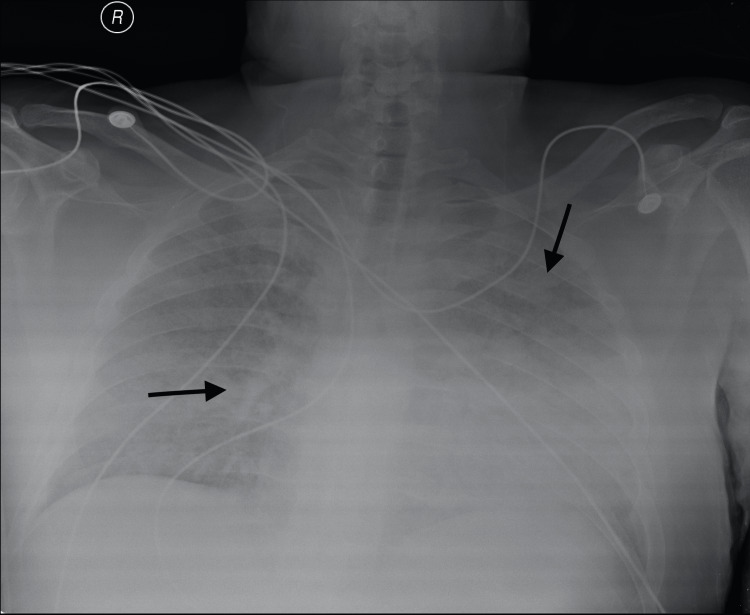
Chest X-ray findings Bilateral multiple alveolar and patchy opacities throughout both lung fields (black arrows) accompanied by complete obliteration of both CP angles.

**Figure 3:**
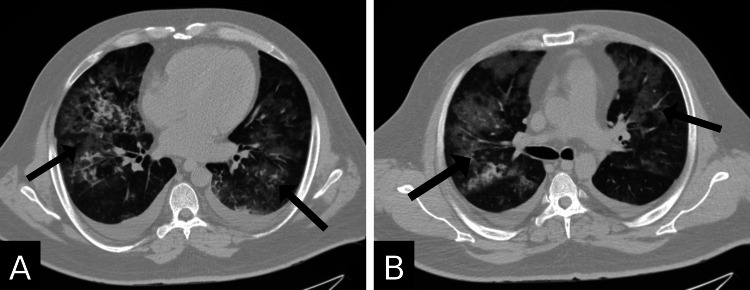
Chest computed tomography scan findings (transverse view; lung window) (A) Lower lung zone: bilateral pleural effusion and multiple scattered areas of ground glass opacity on both lung fields (black arrows). (B) Middle lung zone: bilateral pleural effusion and multiple scattered areas of ground glass opacity on both lung fields (black arrows).

On the 11th day, his kidney function further declined, and his creatinine level reached 4.1 mg/dL. He was identified as having rapidly progressive glomerulonephritis and was started on IV Rituximab. However, the next day, his creatinine function worsened to 5.22 mg/dL. According to the protocol, he then received a session of plasmapheresis, fresh frozen plasma, IV fluids, and IV cyclophosphamide. The hematuria was controlled, and a heparin prophylactic dose was added. Over the next couple of days, the patient received daily plasmapheresis sessions along with a transfusion of two units of fresh frozen plasma. Despite all the ongoing measures being taken, on the 28th day, the patient still had pancytopenia (WBC: 1 × 10^9^/L, RBC: 2.91 × 10^12^/L, and platelet: 39 × 10^9^/L). Ferric carboxymaltose and Darbepoetin alfa were added to address his anemia, and Filgrastim was administered. Three days later, his follow-up labs indicated a WBC: 7.2 × 10^9^/L, RBC: 3.13 × 10^12^/L, and platelet: 40 × 10^9^/L. However, he developed shortness of breath, hypoxia, and hemoptysis, for which he was put on an oxygen mask (5 L/min). Chest imaging findings were suggestive of pulmonary edema and inflammatory changes due to diffuse alveolar hemorrhage (Figures 2-3). Urgent hemodialysis with ultrafiltration of 4 L led to some improvement.

**Figure 4:**
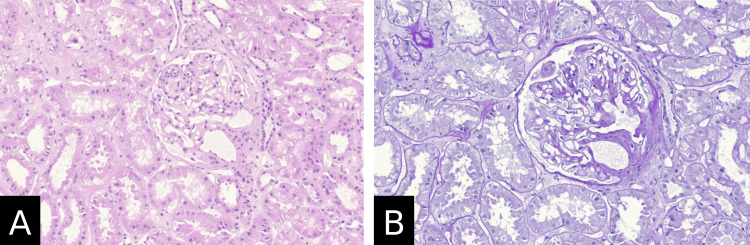
Renal biopsy findings show IgA nephropathy changes (light microscopy) (A) Hematoxylin and eosin stain; original magnification 20× and (B) periodic acid-Schiff stain; original magnification 40×. Crescentic and intracapillary proliferative, mesangioproliferative glomerulonephritis, acute tubular damage, approximately 5-10% tubular atrophy, interstitial fibrosis, and mild arterio-arteriolosclerosis.

**Figure 5:**
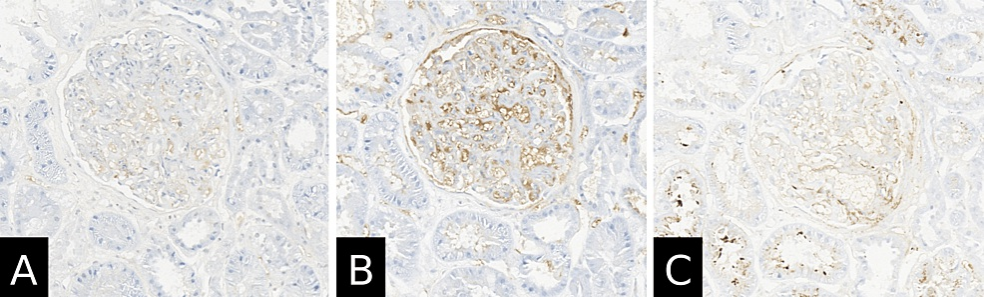
Renal biopsy findings show IgA nephropathy changes (immunohistochemistry) (A) IgA immunohistochemistry; original magnification 40×; (B) IgM immunohistochemistry; original magnification 40×; (C) C3c immunohistochemistry; original magnification 40×; presence of mild deposition in the mesangium.

**Figure 6:**
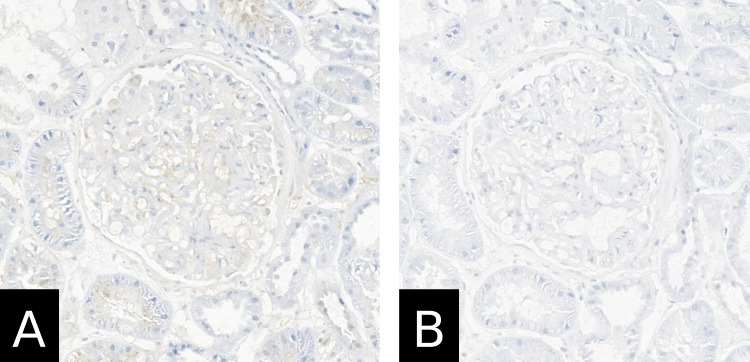
Renal biopsy findings show IgA nephropathy changes (Immunohistochemistry) (A) IgG immunohistochemistry; original magnification 40×; (B) C1q immunohistochemistry; original magnification 40×; no significant staining.

A renal biopsy was done, which showed crescentic and intracapillary proliferative, mesangioproliferative IgA glomerulonephritis, acute tubular damage, approximately 5-10% tubular atrophy, interstitial fibrosis, and mild arterio-arteriolosclerosis (Figure 4). Electron microscopical investigations were consistent with mesangioproliferative IgA glomerulonephritis with severe podocyte damage and no amyloidosis. Immunohistochemistry results showed mild granular mesangial reactivity for IgA, IgM, and C3c (Figure 5) and negative staining for IgG and C1q (Figure 6). The Kongo red stain was negative as well. In the context of clinically reported purpuric skin eruptions, the histopathological findings represent a renal manifestation of HSP and IgAN. Furthermore, molecular genetic analysis of the thiopurine methyltransferase gene (TPMT) was performed, which detected the genotype TPMT*1/*1 (wild type), which is not associated with a pharmacogenetically relevant phenotype.

After more than a month of admission, the patient again developed pancytopenia, markedly deteriorated kidney function, and acute pulmonary edema. 50% replacement plasmapheresis, fresh frozen plasma (FFP), and urgent hemodialysis with UF were done to address these issues. A CT scan of the chest showed mildly regressive minimal basal pleural effusion, progressive ground glass, and regressive small airway disease.

In the second month of admission, the patient received a second dose of rituximab. He was on daily dialysis and received a blood transfusion along with a platelet transfusion for his pancytopenia. He also received Filgrastim due to persistent severe leukopenia and neutropenia. His hematology consultation recommended cryoprecipitate transfusion twice daily as long as his fibrinogen level was low. A week later, the patient received 6 units of platelet transfusions due to severe thrombocytopenia and one packed RBC transfusion due to severe anemia. At the end of the month, azathioprine (50 mg) was added to his medication regimen.

In the third month of his hospital stay, his treatment plan was adjusted to include prednisolone (30 mg). The patient was continuously monitored, labs and kidney functions were followed up, and dialysis was done every alternate day. His urine output started to increase from anuria to 400 ml/day up to 2800 ml/day over a period of two weeks. He was kept on oxygen (3 L/min) along with conservative treatment for his renal failure, which was improving. After being admitted for 80 days, the patient was successfully discharged from the hospital on oxygen and free from dialysis.

## Discussion

IgAN is the most common glomerulonephritis worldwide. It is associated with chronic renal failure, with up to 25% of the patients reaching end-stage renal failure within 20 years of diagnosis [4]. A definitive diagnosis is based on renal biopsy findings, which show granular deposition of IgA and C3 on immunofluorescent staining in an expanded mesangium with foci of segmental proliferative or necrotizing lesions [5]. As in our patient, the presence of IgG is variable, and C1q is typically absent in these patients [5]. Beyond its diagnostic utility in these individuals, renal biopsy has demonstrated its significance in assessing prognosis and predicting the efficacy of specific therapeutic interventions [5]. HSP commonly affects young children as an acute self-limiting entity. Nevertheless, its impact can extend to older children and adults, with an increased propensity for the development of pulmonary hemorrhage in this demographic [3]. Furthermore, late-onset HPS is linked to elevated probabilities of renal involvement and unfavorable prognoses [3,6]. In contrast to HPS, IgAN has demonstrated a higher tendency to manifest concurrently with pulmonary conditions, primarily pulmonary hemorrhage [1]. Nonetheless, the presence of pulmonary hemorrhage has been linked to elevated mortality rates in both cohorts [1,3].

HSP has been linked to IgAN, where both diseases have IgA deposition in the skin and kidneys, respectively, among other tissues. Furthermore, histopathological findings in renal biopsy in both entities are mainly undisguisable due to the shared pathological appearance [4,7]. This shared characteristic poses a challenge in distinguishing the precise contributor to renal dysfunction when both conditions manifest concurrently. Similarly, the pulmonary hemorrhage observed in our patient could arise from the interplay of both conditions or represent the expression of either of them, making it difficult to attribute it to one specific cause. The simultaneous presence of both entities rendered our patient's case intriguing, given that his hospitalization was further complicated by the emergence of acute kidney injury and pulmonary hemorrhage.

While our patient exhibited hemoptysis, it emerged as a late indicator, compounded by the fact that most patients do not experience hemoptysis due to the substantial pulmonary reserve that can accommodate considerable blood volume without overt symptoms [3]. This delay in diagnosis might contribute to higher morbidity and mortality rates. Consequently, a more cautious approach with a lower threshold for conducting bronchoscopy in such cases is recommended.

The pancytopenia experienced by our patient could not be attributed to the immunosuppressive therapy he received, as it occurred prior to its initiation. Despite negative results in his immunological serology, it is plausible that an underlying autoimmune process contributed to this complication. Moreover, this could be an unusual presentation of HSP, as past cases have demonstrated unexplained occurrences, particularly among those with associated renal pathology that might have influenced the progression of pancytopenia. This observation was accompanied by hypocomplementemia, which was also observed in our patient [8]. The resolution of this complication upon the commencement of steroid therapy and bone marrow stimulation suggests a potential association between this manifestation and the disease's pathology. Further research is warranted to delve into the nature of this occurrence within this patient cohort.

Although it is unadvisable to treat HSP patients with steroids, those who develop nephritis or pulmonary hemorrhage have benefited from steroid therapy. In addition, those who had nephritis showed clinical improvement with the addition of cyclophosphamide, azathioprine, or methotrexate. Pulmonary hemorrhage was not successfully managed with steroids alone in the majority of the patients, as they required cyclophosphamide to establish remission and reduce the recurrence rate [3]. Moreover, adjunctive therapeutic options with dialysis, plasmapheresis, and mechanical ventilation could be provided as deemed essential. Given the shared pathomolecular mechanisms, particularly in cases involving pulmonary hemorrhage, the treatment protocol outlined here could be applicable to both HSP and IgAN. Nevertheless, additional research is imperative to establish the most effective therapeutic regimen for this specific patient group, given the persistently elevated mortality rate.

## Conclusions

This case report seeks to enhance our understanding of potential uncommon manifestations of IgAN and HSP. By doing so, it aims to facilitate early recognition and proper management of these atypical presentations. Furthermore, it is important to acknowledge that a subset of patients may experience rapid and severe deterioration, necessitating the implementation of intensive and comprehensive interventions to restore and sustain their well-being. Given this possibility, it is imperative for physicians to maintain heightened awareness and attentiveness, ensuring the timely administration of suitable therapeutic approaches whenever warranted. As an increasing body of evidence accumulates within the literature, it becomes increasingly apparent that additional research is imperative to delineate the optimal approach for enhancing future outcomes within this specific group of patients.
